# The Impact of the Epidemiological Situation Resulting From COVID-19 Pandemic on Selected Aspects of Mental Health Among Patients With Cancer–Silesia Province (Poland)

**DOI:** 10.3389/fpsyg.2022.857326

**Published:** 2022-05-26

**Authors:** Mateusz Grajek, Agnieszka Białek-Dratwa

**Affiliations:** ^1^Department of Public Health, Faculty of Health Sciences Bytom, Medical University of Silesia in Katowice, Bytom, Poland; ^2^Department of Human Nutrition, Faculty of Health Sciences in Bytom, Medical University of Silesia in Katowice, Bytom, Poland

**Keywords:** depression, oncology, COVID-19, lockdown, disease acceptance

## Abstract

**Objective:**

The study aimed to assess the level of disease acceptance as well as the wellbeing and emotions that accompany cancer patients during the COVID-19 pandemic.

**Materials and Methods:**

The study involved 1,000 patients of the oncology centers (Silesia, Poland). The following questionnaires were used for the study: WHO-5–Well-Being Index, BDI–Beck Depression Inventory, disease acceptance scale, and proprietary multiple-choice questions regarding the impact of the epidemic situation on the respondents’ lives so far. The questionnaire study was conducted twice: in March-October 2020 (the beginning of the COVID-19 pandemic in Poland) and March-October 2021 (renewed tightening of sanitary restrictions in Poland). The differences between the given periods were statistically analyzed.

**Results:**

At the time of the study, the respondents felt mainly anxiety related to the possibility of contracting COVID-19, the possibility of infecting a loved one, or staying in quarantine. Anxiety was also caused by the vision of poorer access to health services. Along with the duration of the epidemic, the acceptance of neoplastic disease has decreased and the incidence of depressive symptoms has increased.

**Conclusion:**

The wellbeing of the respondents deteriorated significantly during the pandemic. The respondents, in their daily functioning, were mainly accompanied by anxiety. The risk of depression has increased and the acceptance of the disease has decreased significantly.

## Background

A serious problem of oncology in Poland is the high dispersion of services and the lack of quality control. This primarily affects the way medical services are provided in this sector, the waiting time, and disparities in access to specific. These problems became much more prominent when some hospitals had to be transformed into COVID-19 units. All of this left patients struggling to find their way in the new COVID-19 world (Narodowa-strategia-onkologiczna, 2020). The COVID-19 pandemic is impacting healthcare operations in Poland, particularly oncology. One in three trials or therapies has been canceled due to the pandemic (World Health Organization [WHO], 2020a; Zwrotnik Raka, 2020a). The problems that patients have faced for years in healthcare facilities have also worsened. Such a situation is not indifferent to the mental state of patients. The vision of canceling examinations, and thus aggravating health problems, reflects negatively not only on the wellbeing of patients but also on the acceptance of the disease (Polityka Zdrowotna, 2020). Patients, in many cases, were left without hospital and specialist care.

Pandemic COVID-19 has stirred up a lot of negative emotions since its inception. Both connected with the very fact of the appearance of a pathogen in the public space on such a large scale, as well as controversial issues of introducing more and more new restrictions. Due to the epidemiological situation around the world, many medical sectors have been unable to provide their patients with the support they expected and to meet their treatment deadlines. At the beginning of the pandemic, there was a lack of more information about the potential impact of the pandemic on mental health (Ayubi et al., 2021; Dibble and Connor, 2021; Moghadam et al., 2021). This means that we are ill-prepared to support communities facing the unprecedented COVID-19 pandemic (Huang et al., 2020; Sahin et al., 2020; Kheirvari and Anbara, 2021; Rodrigues-Oliveira et al., 2021). To date, there have been several studies demonstrating the effects of COVID-19 on mental health, mainly in terms of increased worry (Ongaro et al., 2021), anxiety (Petrocchi et al., 2021), health anxiety (Landi et al., 2020), depression, and post-traumatic stress disorder (Preti et al., 2021). The emotional impact of the COVID-19 pandemic on cancer patients has also been extensively described. The authors cite limited access to services and high psychological burden as among the major factors that contributed to anxiety and depression among cancer patients (Wang et al., 2020; Bandinelli et al., 2021; Brivio et al., 2021).

As experience has shown, psycho-oncological support in cancer treatment plays an extremely important role. Patients who receive professional educational, psychological, and/or psychiatric support during their cancer treatment have better treatment outcomes. Psycho-oncological assistance is important at every stage of the disease. The goal of the psycho-oncologist is to improve the quality of life of the patient and their loved ones in the social environment regardless of the severity of cancer. The work of the psycho-oncologist depends on the patient’s communicative and cognitive abilities and his or her willingness to enter into a therapeutic relationship.^1^ Initially, at the time of diagnosis, it helps the patient and their loved ones face the acceptance of the often unexpected and shocking news and helps build motivation for effective treatment (Zwrotnik Raka, 2020b). The COVID-19 pandemic contributed to great difficulties in access, not only to strictly medical services but also to psychological counseling and therapy (World Health Organization [WHO], 2020b). Therefore, it seems reasonable to investigate the scale of ill-being and depressive symptoms among people undergoing oncological treatment.

The following hypotheses about oncology care in the face of a protracted epidemiologic situation seem crucial:

a. There has been a shift in oncology patients within the emotions associated with the COVID-19 pandemic.
b. Oncology patients have experienced a change in general wellbeing related to the COVID-19 pandemic.
c. Oncology patients experienced a change in disease acceptance in relation to pandemic COVID-19.
d. Oncology patients experienced a change in risk of depression in association with pandemic COVID-19.

The purpose of this study was to evaluate the level of acceptance of the disease, assessing the wellbeing and emotions that accompany cancer patients during the COVID-19 pandemic.

First and foremost, the research conducted was to answer four key questions:

A. Did the changing and prolonged epidemiological situation affect respondents’ related experiences (including emotions)?
B. Did the changing and prolonged epidemiological situation worsen the wellbeing of respondents?
C. Did the changing and prolonged epidemiological situation affect the development of depressive symptoms?
D. Has the changing and prolonged epidemiological situation influenced the decrease of acceptance of the disease?

## Materials and Methods

### Test Sample

The questionnaire survey was conducted twice: in March to October 2020 (beginning of the COVID-19 pandemic in Poland; Introduction of restrictions on the postponement of planned treatments in medical facilities)–**T0** and in March to October 2021 (lifting sanitary restrictions in Poland; A return to a partially standard medical work model–reintroducing traditional visits in exchange for telephone counseling.)–**T1**. The same group participated in both study periods.

The study involved 1,000 patients of the oncology centers (Silesia, Poland). The questionnaire was conducted in the office of the oncology coordinators. The oncology coordinator was instructed by his office to carry out a questionnaire among the patients under his care (in Poland, the oncology coordinator is part of the so-called rapid oncology pathway and the first person the patient meets before entering the diagnostic and treatment process in oncology and the person who consults the patient’s condition, therefore the choice of coordinators’ offices was considered the best place to carry out the study).

In total, 44.0% of the patients classified in the study suffered from cancer located in the chest (lung cancer), 32.0% breast cancer; 14% cervical cancer; 10% other causes (including head and neck cancer).

All study participants were informed of the purpose of the study, its anonymity, and were asked to accept the data sharing policy. Information about informed and voluntary participation in the study was at the beginning of the questionnaire.

The study was approved by the Bioethics Committee of the Medical University of Silesia in Katowice (PCN/0022/KB/211/20) in light of the Act on Medical and Dental Professions of December 5, 1996, which includes a definition of medical experimentation. The study participants consciously agreed to participate in the study.

### Study Inclusion and Exclusion Criterion

The initial study group included 1,093 patients; however, due to several factors (significant deterioration in health or death of the respondent between study periods, incorrectly completed questionnaire, or failure to report for a repeat study), the final study group was 1,000 patients.

The criterion for inclusion in the study was: being a patient of oncology centers, cancer disease in different stages/treatment, and no psychological problems in the past that required psychological consultation or psychiatric treatment (no history of psychological help in the form of psychological counseling or psychotherapy related to mood or anxiety disorders), being of legal age (age over 18), informed and voluntary consent to participate in an anonymous study, answer all questions asked by the oncology coordinator. The exclusion criterion was the age under 18, no cancer disease in the medical history, mental illnesses requiring psychological consultation and psychiatric treatment, no consent to participate in the study, no answers to all questions. The above criteria were verified by providing information to the questions asked in the study and based on medical documentation.

### Research Tool

The research tool was a survey questionnaire that consisted of several parts including WHO-5–Good Self-Concept Index developed by the Psychiatric Research Unit of WHO (Topp et al., 2015); BDI–depression scale according to Beck et al. (1961) and Beck and Beck (1972); AIS–Illness Acceptance Scale (Juczyński, 2001); author’s multiple-choice questions about anxiety the impact of the epidemic situation on the respondents’ current life: experiences with COVID-19 in the study patient group (the percentage of people with a given experience was determined); emotions associated with COVID-19 in the study group of patients (distinctions were rated on a scale of 0–10, where 10 represents the highest level of emotion in a given category).

The WHO-5 scale consisted of 5 questions. To obtain a base score, the number of points awarded in each question was summed. A single question was scored from 0 to 5, where 0 meant the worst possible quality of life and 5 meant the best possible quality of life. The point range for the entire scale was 0–25 points. The raw score had to be multiplied by 4 to get the percentage score (%). The percentage score was read according to a scale: 92–100%–feeling very good; 76–91%–good; 56–75%–moderate; 55% and less–bad.^2^ Cronbach’s α coefficient for the normalization sample was 0.92 (Topp et al., 2015).

The BDI included 21 questions about the core symptoms of depression. Questions were scored on a scale from 0 to 3, where 3 meant the highest intensity of a given symptom. The final test score was obtained by summing the number of points obtained for each question and determining the degree of depression according to the following scale: 0–11 no depression; 12–19 mild depression; 20–25; moderate depression; 26 and above–severe depression. Cronbach’s α coefficient for the normalization sample was 0.89 (Beck et al., 1961; Beck and Beck, 1972).

The AIS scale is used to measure the degree of acceptance of the disease–greater acceptance means better adaptation to the disease and less psychological discomfort. The responses were ranked according to the Likert scale, where 1–“strongly agree,” 5–“strongly disagree.” Obtaining the least number of points expresses poor adaptation to the disease. The maximum score is 40 points. Cronbach’s α coefficient for the normalization sample was 0.85 (Juczyński, 2001).

All indicated tools were used in T0 and T1.

### Statistical Analysis

The study was conducted longitudinally. Statistical analysis was performed using the software in Statistica v. 13.3 (StatSoft Inc., Tulsa, OK, United States). Basic descriptive statistics were used in the analysis of the results. Statistical significance was determined using the McNemar chi-square, Wilcoxon and Friedman tests. For dependent trials, the Wilcoxon test was used to determine the differences between the period T0 and T1. In the assessment of the acceptance of the disease, the Friedman test was used due to the presence of many test samples and the specificity of calculating the result. To evaluate the effect size r-Pearson and W-Kendall coefficients was used. For each test, a statistical significance level of *p* = 0.05 was assumed.

## Results

The majority of the respondents were women (64%), the average age was over 47 ± 5 years (18–70 years), most respondents had vocational (35%) and secondary (32%) education, were married (68%), lived in a city (62%) with family (49%), and had offspring (73%). 43% of the respondents supported themselves from their professional work, from retirement–27%, and pension–30%. Generally, it should be stated that this was a group with an average (59%) material situation. The average time from the detection of cancer to the time of the study was 2.5 years. The respondents constituted a group of people with different stages of cancer disease, and also, they varied in terms of the therapy used in their case.

Based on the respondents’ experience of the epidemiological situation related to COVID-19, it can be observed that in the initial phase of the pandemic in Poland the main experience was the need for self-isolation from other people, this phenomenon concerned 72% of the surveyed population. A small percentage of people reported experiences of being infected or suspected of being infected in themselves or someone close to them. Later in the pandemic, it was observed that the other reported experiences with the COVID-19 in the survey increased significantly. In T1, 68% of respondents reported suspected COVID-19 in themselves, 22% were quarantined, 21% were isolated, and 22% were hospitalized for COVID-19, values that were significantly higher than the previous survey period. This difference was statistically significant with Wilcoxon test (*p* = 0.001; *Z* = 12.823; *r* = 0.841)–Table 1.

**TABLE 1:** Experiences with COVID-19 in the study patient group (*N* = 1000).

Experiences with COVID-19	T0		T1		*p*-value
	*X*	*SD*	*X*	*SD*	
Suspected COVID-19 in self	150 (15%)	± 1%	680 (68%)	± 2%	0.001
Self-isolation (as part of prophylaxis)	620 (62%)	± 5%	840 (84%)	± 4%	
Administrative quarantine (as a result of finding COVID-19 in a person in the community)	80 (8%)	± 3%	220 (22%)	± 4%	
Isolation (following a COVID-19 diagnosis)	40 (4%)	± 2%	210 (21%)	± 3%	
Hospitalization (after COVID-19 diagnosis)	40 (4%)	± 2%	220 (22%)	± 2%	

For emotions directly related to the COVID-19 pandemic, respondents most frequently reported feelings of anxiety about infecting themselves or someone around them, anxiety about quarantine, isolation, or hospitalization resulting from contracting COVID-19, and indirectly, feelings of anxiety related to loss of the source of income and anxiety about poorer access to health services, social misinformation, and lack of contact with loved ones. It was observed that the mentioned factors directly related to COVID-19 increased by an average of 3.08 points (on a 10-point scale), suggesting that negative emotions related to COVID-19 were higher during the T1 study period than in T0 (*p* = 0.023; *Z* = 10.411; *r* = 0.623). A similar situation was observed for intermediate factors such as: “anxiety about poorer access to services” (up 6.50 points) and “anxiety about public misinformation” (up 4.04 points). For these two distinguishing factors, the differences were also statistically significant with Wilcoxon test (*p* = 0.001; *Z* = 11.825; *r* = 0.662)–Table 2.

**TABLE 2:** Emotions associated with COVID-19 in the study group of patients (*N* = 1000).

Emotions of COVID-19*	T0			T1			Difference	*p*-value
	*X*	*SD*	*min-max*	*X*	*SD*	*min-max*		
**Emotions directly related to COVID-19**								
Anxiety about COVID-19 infection in yourself	6.22	± 1.28	2–8	8.68	± 0.96	4–10	↑2.46	0.023
Anxiety about COVID-19 infection in a love one	4.34	± 1.02	1–6	6.62	± 1.44	3–8	↑2.28	
Anxiety about quarantining	4.62	± 1.32	1–6	6.98	± 1.14	3–8	↑2.36	
Anxiety about the isolation	4.12	± 0.98	1–6	8.24	± 0.92	4–10	↑4.12	
Anxiety about the hospitalization	4.38	± 1.06	1–6	8.58	± 1.34	4–10	↑4.20	
**Emotions indirectly related to COVID-19**								
Anxiety of losing employment	2.22	± 1.08	0–5	2.56	± 1.56	0–5	↑0.34	0.09
Anxiety about poorer access to medical services	2.32	± 1.12	0–5	8.82	± 1.22	3–10	↑6.50	0.001
Anxiety about social misinformation	2.84	± 1.24	0–5	6.90	± 0.88	2–8	↑4.04	0.001
Anxiety about not being able to contact family/friends	2.40	± 0.92	0–5	2.68	± 0.96	0–5	↑0.28	0.11

*All distinctions were rated on a scale of 0–10, where 10 represents the highest level of emotion in a given category.

Using the WHO-5 scale, the level of wellbeing of the study patients was assessed. It was shown that compared to T0, the wellbeing decreased by 5.28 points (21.12%) in T1. The result obtained during the implementation of the study should be interpreted as a decrease in wellbeing between T0 and T1 with Wilcoxon test (*p* = 0.012; *Z* = 14.346; *r* = 0.897)–Table 3.

**TABLE 3:** Results of WHO-5 scale in the studied group of patients (*N* = 1000).

In the last 2 weeks, I have felt:*	T0			T1			Difference	*p*-value
	*X*	*SD*	*min-max*	*X*	*SD*	*min-max*		0.012
Happy and in a good mood	3.34	± 0.98	0–5	1.96	± 0.64	0–4	↓1.38	
Calm and relaxed	2.08	± 0.82	0–5	1.58	± 0.98	0–4	↓0.50	
Active and energetic	2.14	± 0.78	0–4	1.44	± 0.74	1–4	↓0.70	
Fresh and well-rested	2.24	± 1.12	0–5	1.26	± 0.88	0–4	↓0.90	
My life was filled with things that interested me	2.60	± 0.90	0–5	0.88	± 0.44	0–4	↓1.72	
Total points	12.40	± 0.92	7–18	7.12	± 0.74	5–15	↓5.28	

*All discriminators were rated on a scale of 0–5, where 5 meant that a particular feeling had accompanied the subject for at least 2 weeks before the study period.

The trend noted for the wellbeing scale was also reflected in the level of depressive symptoms in the study population. It was shown that in the initial phase of the study (T0) the absence of depression characterized 71% of the subjects (68% of women, 75% of men). Symptoms of mild depression were demonstrated in 25% of the subjects and moderate depression in 6%. There were no subjects with severe depressive symptoms during this period. In T1, there was a decrease in those showing no depressive symptoms (by 11%). There was an increase of 6% in those with mild to moderate depression and 1% for severe depression (*p* = 0.001; *Z* = 10.072; *r* = 0.684)–Table 4 (Wilcoxon test).

**TABLE 4:** BDI test results in the study group of patients (*N* = 1000).

Study period	State of depression				*p*-value
	No depression	Mild	Moderate	Hard	
T0	710 (71%)	250 (25%)	40 (4%)	0 (0%)	0.001
T1	600 (60%)	310 (31%)	80 (8%)	10 (1%)	
Difference	↓11%	↑6%	↑6%	↑1%	

Acceptance of the disease was measured using the AIS scale. Based on the results of this scale it was stated that in the period when the epidemiological situation in Poland was still developing and the number of cases did not exceed 1,000 new cases per day, the acceptance of the disease among the examined patients scored 28.94 points on average (out of 40 possible to obtain–which indicated high acceptance of the disease). The repeated survey conducted in T1 showed a decrease in the number of points about the survey conducted in T0 by 6.83 points on average (a 17% decrease in point values among patients), and thus a worse acceptance of the disease in the period of the dynamic increase in the number of cases and the imposition of the greatest restrictions related to movement and access to medical services. There was a decrease in values for all disease acceptance traits with Friedman test (*p* = 0.003; *X*^2^ = 10.442; *W* = 0.628). Detailed data are presented in Table 5.

**TABLE 5:** AIS scale scores in the study group of patients (*N* = 1000).

AIS*	T0			T1			Difference	*p*-value
	*X*	*SD*	*min-max*	*X*	*SD*	*min-max*		
I have trouble adapting to the limitations imposed by my illness	4.48	± 0.90	1–5	3.24	± 1.12	1–5	↓1.24	0.003
I am unable to do the things I enjoy most because of my condition	4.12	± 1.30	1–5	3.74	± 1.24	1–5	↓0.38	
My illness sometimes makes me feel unnecessary	3.98	± 0.92	1–5	3.26	± 0.96	1–5	↓0.30	
Health problems make me more dependent on others than I want to be	3.56	± 1.08	1–5	2.98	± 0.88	1–5	↓0.58	
Illness makes me a burden to my family and friends	3.82	± 1.62	1–5	2.54	± 1.06	1–5	↓1.28	
My health makes me feel less than a full human being	2.78	± 1.02	1–5	1.98	± 1.18	1–5	↓0.80	
I will never be as self-sufficient as I want to be.	3.06	± 1.26	1–5	2.02	± 0.86	1–5	↓1.04	
I think people around me are often embarrassed by my illness	3.14	± 1.48	1–5	2.36	± 1.02	1–5	↓0.78	
Total points	28.94	± 1.20	5–40	22.12	± 1.04	5–40	↓6.82	

*All distinguishing factors were rated on a scale of 1–5. where 5 indicates the highest level of disease acceptance within a given distinguishing factor.

## Discussion

People suffering from cancer are a special group of patients in whose case not only the correctness of the diagnosis and the fact of commencing treatment but also the speed of initiating the whole therapeutic procedure is of significance. Depending on the type, cancer has different rates of progression, yet even a short delay in treatment can cause significant progression of the cancer process and irreversible changes in the body that could have been avoided if therapy had been started promptly. During the COVID-19 pandemic, access to oncology services was hampered. with many follow-up appointments and scheduled treatments canceled or rescheduled. In addition, specialist consultations were carried out using remote communication methods and techniques, which also did not have a positive effect on the patient medical staff relationship. The procedures introduced were aimed at the safety of patients, in whom additional infection could result in serious complications or even death. Moreover, many hospitals were transformed into one-named hospitals (specialized in COVID-19), which also constituted a barrier to planned medical interventions and specialist care. The whole situation affected the psychological condition of patients. Previous studies (Gunnell et al., 2020; Holmes et al., 2020; Sahin et al., 2020; Ayubi et al., 2021; Dibble and Connor, 2021; Kheirvari and Anbara, 2021; Moghadam et al., 2021; Rodrigues-Oliveira et al., 2021) have shown that the development of epidemic is of great public health importance, COVID-19 should be counted among the factors that bring sudden changes in the mental health of the population, which may be due to the fact of dynamic development of this disease. dramatic media coverage and own experience. It has been shown that the sudden appearance of such a large stressor causes a decrease in wellbeing, anxiety, and even psychotic disorders. which in extreme cases can lead to suicidal thoughts and suicidal actions (Li J. et al., 2020). It has also been shown that the lack of psychotherapeutic interventions promotes the transformation of these symptoms into serious psychiatric disorders such as depression. anxiety disorders or post-traumatic stress disorder (Li W. et al., 2020).

As shown in the study, self-reported emotions such as anxiety about COVID-19 infection and anxiety about social insecurity and difficult access to services were the most important emotions accompanying patients during the study period. It should also be noted that the level of perceived anxiety and anxiety in each aspect increased during the second study period (in T1) compared to the first assessment (in T0). This may be related to the protracted duration of the pandemic, the prolonged waiting time for oncology procedures, the rapid increase in the number of patients (especially those burdened with other diseases, including cancer), and media reports that reported a lack of places for new patients in hospitals. an increasing number of deaths, and the inability to perform certain medical procedures. The obtained results prove that the development of the pandemic still causes many negative changes in the level of wellbeing, which indicates the still unfinished process of adaptation to new environmental conditions. Interestingly the largest increase in the anxiety of all aspects examined was associated with anxiety of poorer access to medical services (an increase from 2.32 points in T0 to 8.82 points in T1) with a much smaller increase in feelings of anxiety from worrying about one’s illness (6.22 points in T0 and 8.68 points in T1). These results may be related to the presence of cancer. Based on them. it can be concluded that respondents at the beginning of the pandemic showed more anxiety related to the possibility of contracting COVID-19, while at the time of the re-survey, the greatest anxiety was related to the delay of treatment of the cancer process and the possible consequences of its absence. Public misinformation also appears to be a major stress factor (increasing from 2.84 points to 6.90 points), making it difficult to separate information that has been confirmed and accepted by researchers and clinicians from widespread lies about the pandemic and information intended to sensationalize that has no basis in reality.

Among the most important factors that affect the quality of life is mental wellbeing. Its disruption negatively affects daily activities and functioning in society. It has been shown that diseases such as depression or anxiety disorders negatively correlate with the quality of life (Kim et al., 2015). It has also been proven that the development of depression among patients with cancer worsens prognosis. increases overall mortality. impairs daily functioning and decreases overall enjoyment of life (Ardebil et al., 2013; Fujisawa et al., 2016). According to epidemiological data, depressive syndromes occur in 20–40% of cancer patients. of which 1/3 of patients are diagnosed with cancer. The percentage of depressive states decreases to 15% in cancer patients during the remission period, to increase again up to 50% during relapse (Skalij et al., 2016). In our study, we found that the mean number of points corresponding to the patients’ wellbeing assessment at the beginning of the study indicated moderate wellbeing, while at the time of the repeat survey, the mean had dropped by as much as 5.28 points according to the WHO-5 scale. indicating a low level of wellbeing in T1. A particular difference between T0 and T1 was noted in the assessment of pursuing passions and interests. Respondents reported that their lives were far less filled with things they were interested in, In the repeat survey respondents also indicated that they were less often in good wellbeing. Significant development of depressive symptoms was also observed (31% of respondents showed symptoms of mild depression and 8% of respondents showed symptoms of moderate depression at the last stage of the study) with a simultaneous decrease in acceptance of the disease. The results obtained in our study were slightly higher than the diagnosis of depression among lung cancer patients in Vietnam (26.4%) (Khue et al., 2019). However, it seems that these differences may be due to the study period–in our work, the study was conducted during the pandemic period, which is an additional risk factor for depression, the study by Khue et al. (2019) was conducted before the pandemic. Despite these minor differences. it is important to recognize that cancer patients should be provided with appropriate psychological support and, if necessary. psychiatric treatment. One of the most effective therapeutic approaches is cognitive-behavioral psychotherapy (Li J. et al., 2020). Cognitive-behavioral therapy for patients with cancer helps to identify stress levels and modify false beliefs, reduce or eliminate symptoms of psychological distress, learn effective ways to cope with difficult situations, and eliminate physical symptoms such as insomnia or chronic fatigue (Li J. et al., 2020).

Acceptance of the disease is one of the key factors influencing the therapeutic process (Kozieł et al., 2016). It is a long-term process, the task of which is to adapt the patient to the fact of the presence of chronic or incurable disease. The level of disease acceptance depends on many factors, e.g., the way of treatment, prognosis, symptoms, complications, own psychological resources, and social support (Kozieł et al., 2016; Qiu et al., 2020). The higher the value of this index. the more the patient shows greater participation in the therapeutic process and feels less discomfort due to the presence of the disease. It has also been shown that a high degree of acceptance of the disease increases the overall satisfaction with life and prolongs the survival time, even when there is an incurable disease (Chabowski et al., 2017; Cipora et al., 2018; Qiu et al., 2020). In our study. it was shown that as the pandemic process is prolonged, the level of acceptance of the disease decreases. A particular decline in acceptance was shown in four categories: difficulty in adapting to the limitations caused by the disease, the feeling of being a “burden” to family and friends, the feeling of being an incomplete human being, and the fear of self-sufficiency in the future. Decreased acceptance of the disease is associated with increased depressive symptoms, lowered wellbeing, and anxiety about the future. Moreover, the results obtained in our study fit Beck’s triad (depressive triad), i.e., the occurrence of negative thoughts about oneself. negative thoughts about the environment and the future, which suggests the need for a deeper assessment of the mental state of the subjects.

It should be considered that the results obtained in our work confirm and complement other studies (Schepisi et al., 2019; Li W. et al., 2020; Swainston et al., 2020) that described the impact of the COVID-19 pandemic on cancer patients. It can be suspected that current arrangements in the healthcare system, resulting directly from the pandemic, are a likely determinant of poorer cancer outcomes. Suspension of some oncological procedures, delays in diagnostic tests, difficult contact with medical personnel, and uncertainty about the future are conducive to the occurrence of increased anxiety, depressive disorders, and lower acceptance of the disease, which results in deterioration of mental state among this group of patients. We urge that appropriate procedures be developed and implemented to ensure that even in the event of a pandemic, patients with cancer have uninterrupted access to healthcare. We also urge that, given the impact of severe stressors on mental health. real psychological support is provided to this group of patients.

Among the important advantages of the study conducted is undoubtedly the large study group, which also carries some risks. In the analyses, we did not take into account the stage of cancer, because the main assumption of the study was to see if there are indications of worsening wellbeing and risk of depression in the study population. In future studies, it is planned to pay attention to the above aspect to conduct detailed statistical analyses. Another undoubted strength of the study was the contact with the patient. The coordinators of the study had direct access to the patients, so it was easy to avoid mistakes when completing the questionnaire, inaccuracies associated with poor understanding of the questions. This resulted in a high return rate. The disadvantage of the method used, which should be noted, is undoubtedly the generalized questionnaire, which did not take into account the specific needs of people belonging to a particular group of oncological patients. In future studies, more specific questions should be formulated to identify possible mental health problems of cancer patients.

## Conclusion

Wellbeing of the respondents deteriorated significantly during the COVID-19 pandemic, with respondents most often indicating that emotions such as fear of infection and anxiety about the future of necessary anti-cancer therapy dominated their lives. It was shown that the risk of depression significantly increased among the respondents. At the same time, acceptance of the disease decreased among the subjects, which also influenced the scores obtained on the wellbeing and depression scales. Nevertheless, this theme should be further observed and studied in detail. The experiences of cancer patients with COVID-19 changed as the epidemiological situation in Poland evolved. Initially, self-isolation and isolation were observed, resulting in a small number of respondents who were ill with COVID-19. Subsequently, the study found that more than half of respondents had suspected COVID-19, and one-fifth of patients had undergone COVID-19-related quarantine or isolation.

## Data Availability Statement

The original contributions presented in the study are included in the article/supplementary material, further inquiries can be directed to the corresponding author.

## Ethics Statement

This study was approved by the Bioethics Committee of the Medical University of Silesia in Katowice (PCN/0022/KB/211/20) in light of the Act on Medical and Dental Professions of December 5, 1996, which includes a definition of medical experimentation. The study participants consciously agreed to participate in the study.

## Author Contributions

MG: idea, methods, and writing. AB-D: statistics, discussion, and review. Both authors contributed to the article and approved the submitted version.

## Conflict of Interest

The authors declare that the research was conducted in the absence of any commercial or financial relationships that could be construed as a potential conflict of interest.

## Publisher’s Note

All claims expressed in this article are solely those of the authors and do not necessarily represent those of their affiliated organizations, or those of the publisher, the editors and the reviewers. Any product that may be evaluated in this article, or claim that may be made by its manufacturer, is not guaranteed or endorsed by the publisher.

## Footnotes

1
https://www.frontiersin.org/articles/10.3389/fpsyg.2021.662777/full

2
http://www.psychiatriapolska.pl/uploads/onlinefirst/Grajek_PsychiatrPolOnlineFirstNr258.pdf
